# Supplementary material for: Detection of apoptosis and matrical degeneration within the intervertebral discs of rats due to passive cigarette smoking
Source: PLoS One. 2019 Aug 27;14(8):e0218298. doi: 10.1371/journal.pone.0218298 (PMC6711513; doi:10.1371/journal.pone.0218298)
Supplement: S1 Fig — (A) Immunohistochemistry for type II collagen. (B) Immunohistochemistry for aggrecan. (C) Safranin O staining. The upper and lower sides of each panel represent the cranial and caudal directions, respectively. The left and right sides of each panel represent the ventral and dorsal directions, respectively. The positive NP area ratio was the ratio of the positive area to the entire NP, and the positive AF area ratio was the ratio of the positive area to the dorsal AF with a diameter of 300 μm. The CEP was divided into the peripheral and central regions, and the positive area percentage was calculated in each region. The bar indicates 200 μm. (PDF) [file pone.0218298.s001.pdf]

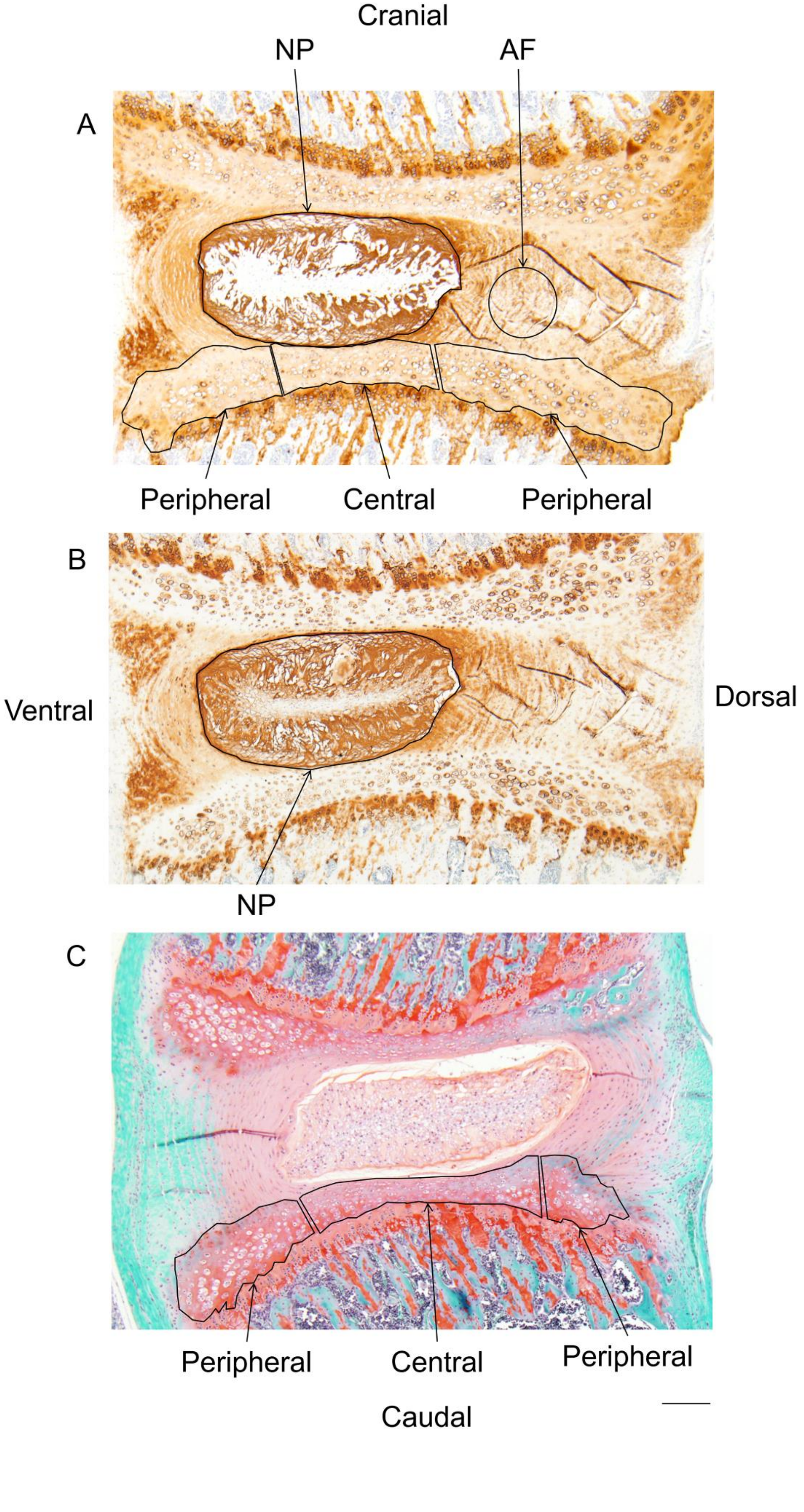
Cranial
NP
AF
A
Peripheral
Central
Peripheral
B
Ventral
Dorsal
NP
C
Peripheral
Central
Peripheral
Caudal

S1 Fig. Measurement area for histological examination.

(A) Immunohistochemistry for type II collagen. (B) Immunohistochemistry for aggrecan. (C) Safranin O staining. The upper and lower sides of each panel represent the cranial and caudal directions, respectively. The left and right sides of each panel represent the ventral and dorsal directions, respectively. The positive NP area ratio was the ratio of the positive area to the entire NP, and the positive AF area ratio was the ratio of the positive area to the dorsal AF with a diameter of 300 μm. The CEP was divided into the peripheral and central regions, and the positive area percentage was calculated in each region. The bar indicates 200 μm.
