# Supplementary material for: Detection of apoptosis and matrical degeneration within the intervertebral discs of rats due to passive cigarette smoking
Source: PLoS One. 2019 Aug 27;14(8):e0218298. doi: 10.1371/journal.pone.0218298 (PMC6711513; doi:10.1371/journal.pone.0218298)
Supplement: S2 Fig — (A) Type II collagen and Mmp13 mRNA. (B) Aggrecan, Mmp3, and Adamts4 mRNA. Two IVD were excised from each rat and homogenized in TRIZOL (Invitrogen, Carlsbad, USA) to extract total RNA. The extracted RNA was treated with DNase I, and then reacted with random hexamer primers and Prime Script reverse transcriptase (TAKARA, Kyoto, Japan) at 30°C for 10 min, 45°C for 60 min, and 70°C for 15 min to synthesize cDNA. Using the TaqMan Gene Expression Assay (Applied Biosystems, Foster, USA), PCR was performed according to the manufacturer’s instructions using a Rotor-Gene 6000 real-time analyzer (Corbett Life Science QIAGEN, Alabama, USA). 18S rRNA was measured as an endogenous control for correction of the gene expression levels. For quantification, the absolute quantification method was employed, in which a calibration curve was prepared for each gene from 5-fold serial dilutions using cDNA with the highest expression level as the standard. The measured expression level of each gene was divided by the measured 18S rRNA expression level to calculate the normalized value. This normalized value was compared between the groups. The significance of the differences was analyzed using the Mann-Whitney U test. (PDF) [file pone.0218298.s002.pdf]

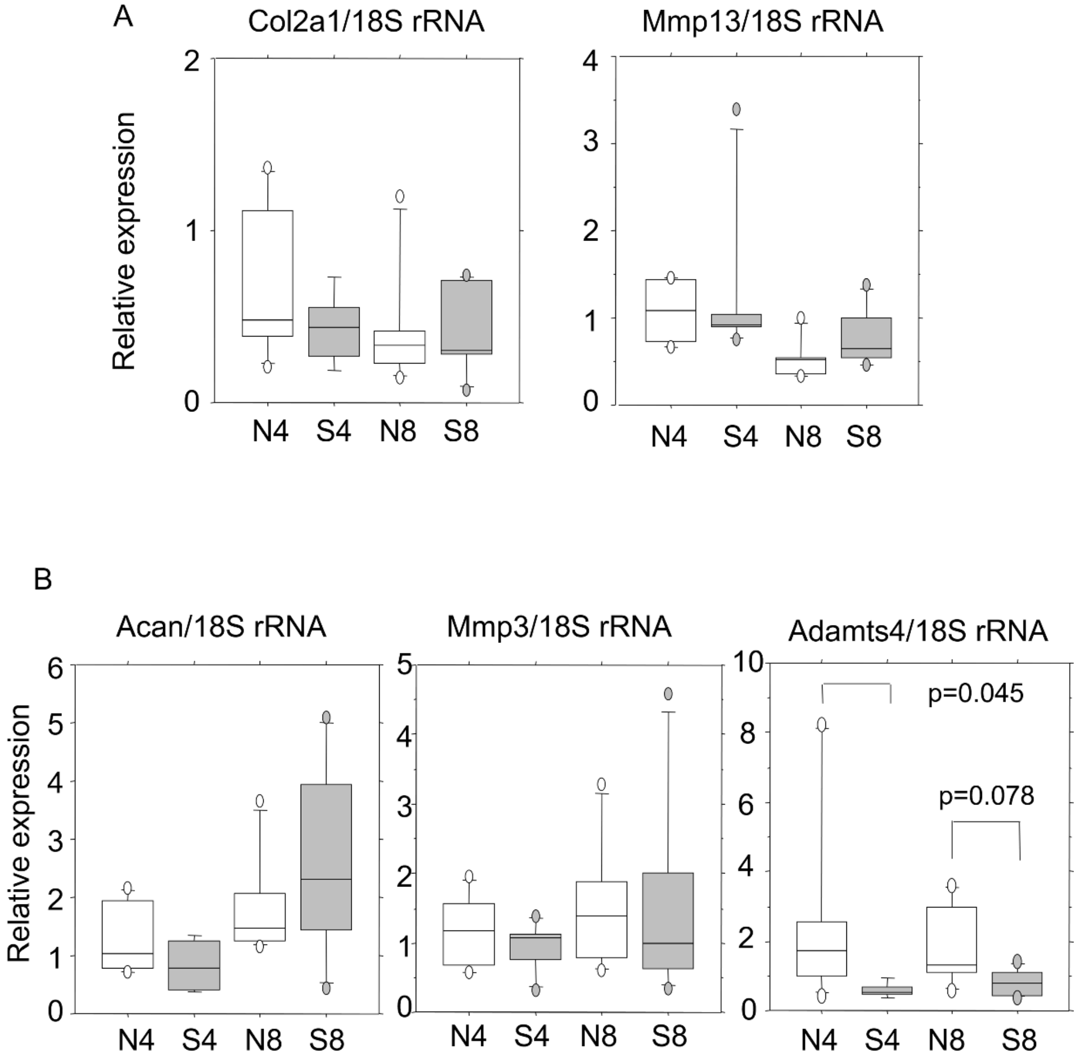


**S2 Fig. Quantitative mRNA analysis.**

(A) Type II collagen and Mmp13 mRNA. (B) Aggrecan, Mmp3, and Adamts4 mRNA. Two IVD were excised from each rat and homogenized in TRIZOL (Invitrogen, Carlsbad, USA) to extract total RNA. The extracted RNA was treated with DNase I, and then reacted with random hexamer primers and Prime Script reverse transcriptase (TAKARA, Kyoto, Japan) at 30°C for 10 min, 45°C for 60 min, and 70°C for 15 min to synthesize cDNA. Using the TaqMan® Gene Expression Assay (Applied Biosystems, Foster, USA), PCR was performed according to the manufacturer’s instructions using a Rotor-Gene 6000 real-time

analyzer (Corbett Life Science QIAGEN, Alabama, USA). 18S rRNA was measured as an endogenous control for correction of the gene expression levels. For quantification, the absolute quantification method was employed, in which a calibration curve was prepared for each gene from 5-fold serial dilutions using cDNA with the highest expression level as the standard. The measured expression level of each gene was divided by the measured 18S rRNA expression level to calculate the normalized value. This normalized value was compared between the groups. The significance of the differences was analyzed using the Mann-Whitney U test.
