# Supplementary material for: Detection of apoptosis and matrical degeneration within the intervertebral discs of rats due to passive cigarette smoking
Source: PLoS One. 2019 Aug 27;14(8):e0218298. doi: 10.1371/journal.pone.0218298 (PMC6711513; doi:10.1371/journal.pone.0218298)
Supplement: S3 Fig — Left and right panels represent low and high magnification, respectively. Bars indicate 1 mm and 200 μm, respectively. N4, non-smoking control for 4 weeks; S4, passive smoking for 4 weeks; N8, non-smoking control for 8 weeks; S8, passive smoking for 8 weeks. (PDF) [file pone.0218298.s003.pdf]

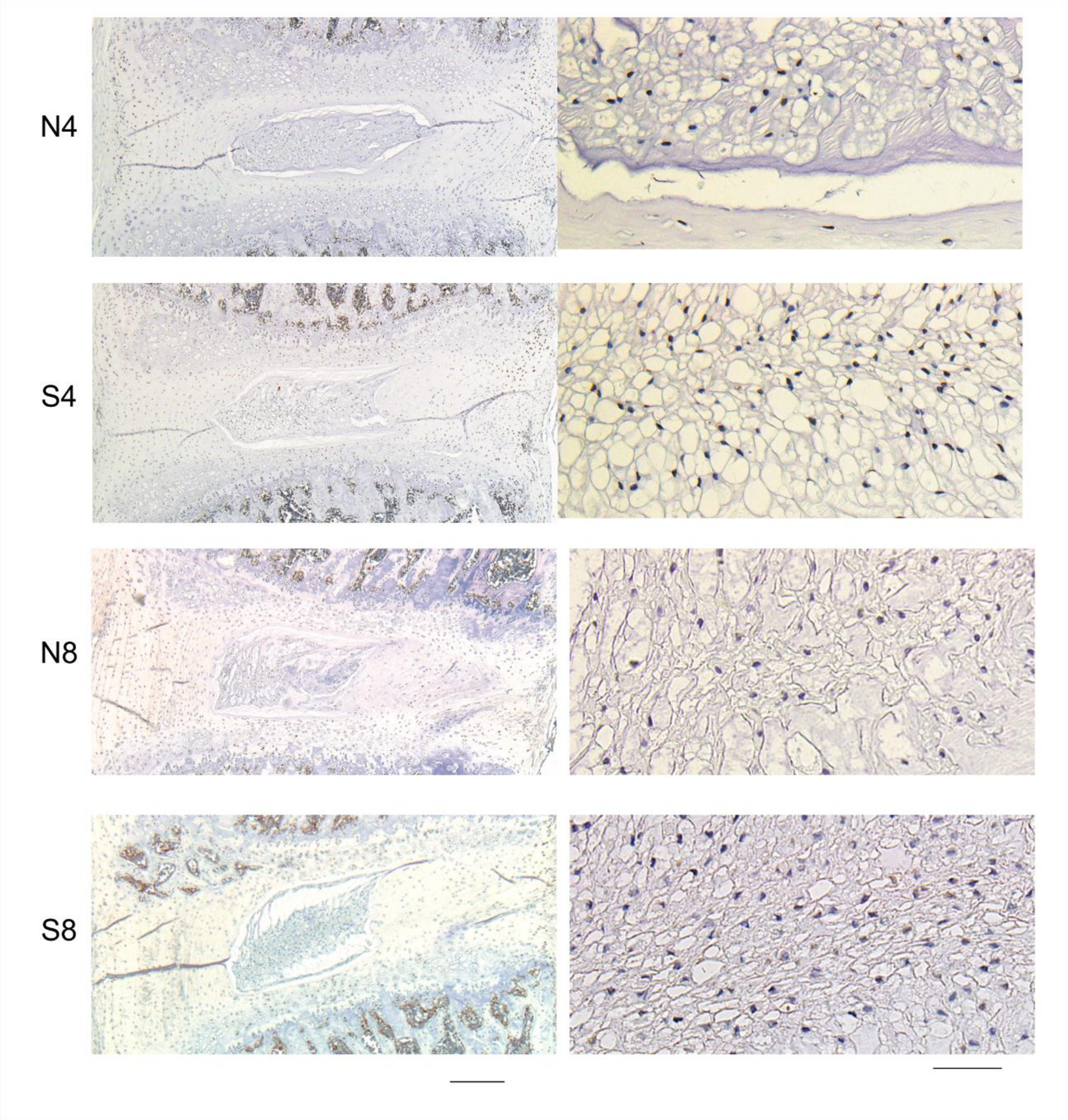

S3 Fig. Immunostaining for ssDNA in the NP and AF.

Left and right panels represent low and high magnification, respectively. Bars indicate 1 mm and 200 μm, respectively. N4, non-smoking control for 4 weeks; S4, passive smoking for 4 weeks; N8, non-smoking control for 8 weeks; S8, passive smoking for 8 weeks.
