# Supplementary material for: Detection of apoptosis and matrical degeneration within the intervertebral discs of rats due to passive cigarette smoking
Source: PLoS One. 2019 Aug 27;14(8):e0218298. doi: 10.1371/journal.pone.0218298 (PMC6711513; doi:10.1371/journal.pone.0218298)
Supplement: S4 Fig — (A) Cleavage sites of caspase 1 and caspase 3 in rat β-actin. The forty-two-kDa β-actin is cleaved by caspases-1 at 2 aspartic acid (Asp) residues at positions 11 and 244, producing a 29-kDa fragment. It is also cut at Asp 244 by caspase 3 to produce a 32-kDa fragment. The thick bar from amino acid residues 1 to 100 indicates the epitope of the anti-β-actin antibody used in this study. (B) Immunoblot analysis of β-actin from the IVD (NP and AF). Three IVD from each of 5 rats were combined, and protein was extracted. The IVD were mechanically ground using a mortar, cooled in liquid nitrogen, and extracted with shaking in 1 ml of guanidine hydrochloride extraction solution (4 M guanidine HCl, 50 mM sodium acetate, 65 mM DTT, 10 mM EDTA, Complete Mini Protease Inhibitor Cocktail (Roche), pH 8.5) at 4°C overnight. After centrifugation at 30,000(x g) for 5 min, precipitated collagen fibers were removed, and the supernatant was centrifuged again to remove macromolecular proteins that were 100 kDa or larger using a 100 kDa molecular weight cut off centrifugal filter (Millipore, CA, USA). The filtrate was used as the protein extract. The extract was mixed with 9 volumes of 100% ethanol to precipitate any protein. The precipitate was washed and resolved with 1xSDS-PAGE loading buffer. Forty μg of protein was applied to a 12.5% polyacrylamide gel and electrophoresed, followed by blotting onto a nitrocellulose membrane using the iBlot Dry Blotting System (Carlsbad, USA). The membrane was blocked with 5% skim milk/PBS at room temperature for one hour and then reacted with 0.3 μg/ml of mouse monoclonal anti-β-actin antibody (Abcam, Cambridge, UK) at room temperature for one hour, followed by reaction with 0.02 μg/ml of HRP-conjugated anti-mouse IgG goat antibody at room temperature for 30 minutes. Chemiluminescence was induced using ECL Advance (GE Healthcare, Buckingham, UK) and detected using Light-Capture (Atto, Tokyo, Japan). Two bands corresponding to β-actin, 42- and 32-kD [file pone.0218298.s004.pdf]

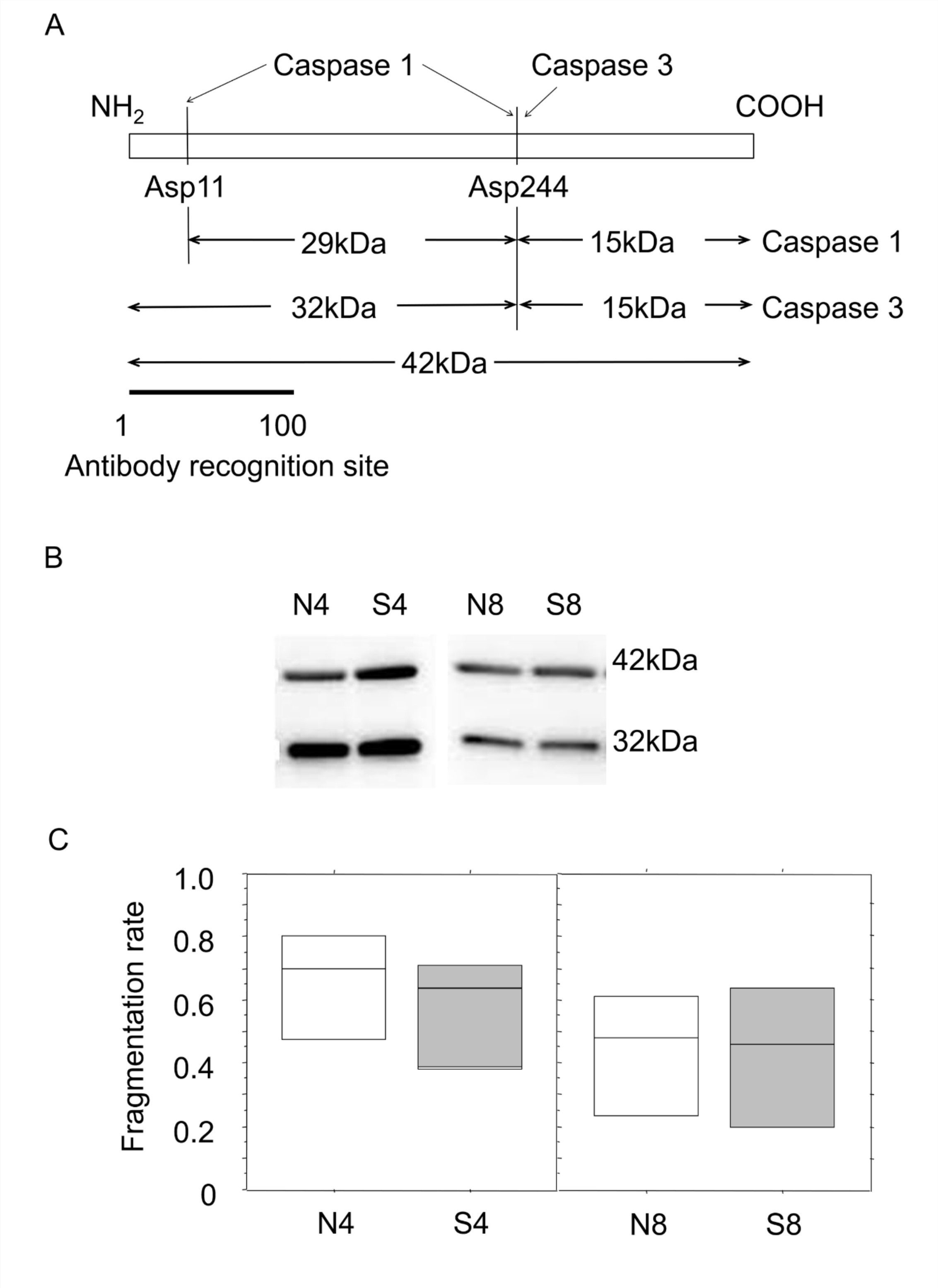


**S4 Fig. Fragmentation of β-actin.**

(A) Cleavage sites of caspase 1 and caspase 3 in rat β-actin. The forty-two-kDa

β-actin is cleaved by caspases-1 at 2 aspartic acid (Asp) residues at positions 11 and 244, producing a 29-kDa fragment. It is also cut at Asp 244 by caspase 3 to produce a 32-kDa fragment. The thick bar from amino acid residues 1 to 100 indicates the epitope of the anti-β-actin antibody used in this study. (B) Immunoblot analysis of β-actin from the IVD (NP and AF). Three IVD from each of 5 rats were combined, and protein was extracted. The IVD were mechanically ground using a mortar, cooled in liquid nitrogen, and extracted with shaking in 1 ml of guanidine hydrochloride extraction solution (4 M guanidine HCl, 50 mM sodium acetate, 65 mM DTT, 10 mM EDTA, Complete Mini Protease Inhibitor Cocktail (Roche), pH 8.5) at 4°C overnight. After centrifugation at 30,000*(x g)* for 5 min, precipitated collagen fibers were removed, and the supernatant was centrifuged again to remove macromolecular proteins that were 100 kDa or larger using a 100 kDa molecular weight cut off centrifugal filter (Millipore, CA, USA). The filtrate was used as the protein extract. The extract was mixed with 9 volumes of 100% ethanol to precipitate any protein. The precipitate was washed and resolved with 1xSDS-PAGE loading buffer. Forty µg of protein was applied to a 12.5% polyacrylamide gel and electrophoresed, followed by blotting onto a nitrocellulose membrane using the iBlot Dry Blotting System (Carlsbad, USA). The membrane was blocked with 5% skim milk/PBS at room temperature for one hour and then reacted with 0.3 µg/ml of mouse monoclonal anti-β-actin antibody (Abcam, Cambridge, UK) at room temperature for one hour, followed by reaction with 0.02 µg/ml of HRP-conjugated anti-mouse IgG goat antibody at room temperature for 30 minutes. Chemiluminescence was induced using ECL Advance (GE Healthcare, Buckingham, UK) and detected using Light-Capture (Atto, Tokyo, Japan). Two bands corresponding to β-actin, 42- and 32-kDa, were also detected in the IVD in the non-smoking control groups, suggesting that physiological cleavage of β-actin by caspase 3 occurs in the normal rat IVD. N4,

non-smoking control for 4 weeks; S4, passive smoking for 4 weeks; N8, non-smoking control for 8 weeks; S8, passive smoking for 8 weeks. (C) Quantification of immunoblotting for β-actin. Immunoblot signals were subjected to molecular weight measurement and quantitative analysis using CS Analyzer 2.0 (Atto) and MultiGauge (FUJIFILM, Tokyo, Japan), respectively. The proportion of β-actin fragmentation by caspase 3 was calculated. Passive cigarette smoking did not induce any change in the fragmentation rate.
