# Supplementary material for: Detection of apoptosis and matrical degeneration within the intervertebral discs of rats due to passive cigarette smoking
Source: PLoS One. 2019 Aug 27;14(8):e0218298. doi: 10.1371/journal.pone.0218298 (PMC6711513; doi:10.1371/journal.pone.0218298)
Supplement: S5 Fig — * indicates significant decrease in body weight gain in smoking rats (p<0.05 by Mann-Whitney U test). (PDF) [file pone.0218298.s005.pdf]

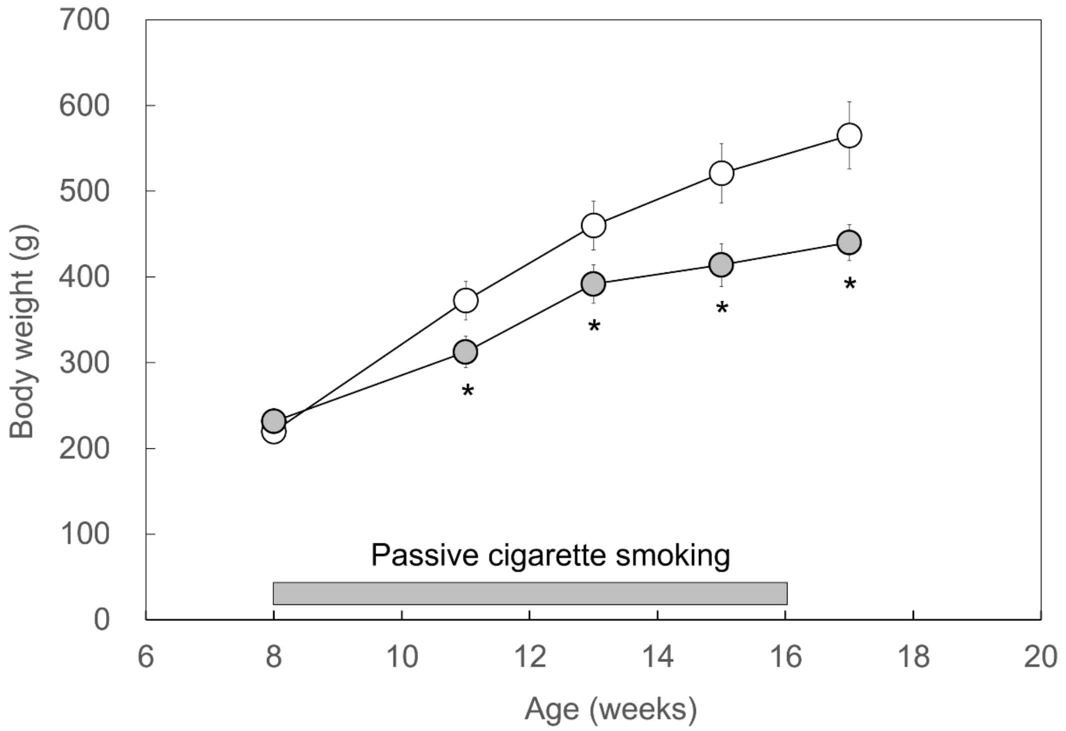

S5 Fig. Rat body weight during passive cigarette smoking (grey) compared with that of non-smoking control (white).

* indicates significant decrease in body weight gain in smoking rats ($p<0.05$ by Mann-Whitney U test).
