# Supplementary material for: Detection of apoptosis and matrical degeneration within the intervertebral discs of rats due to passive cigarette smoking
Source: PLoS One. 2019 Aug 27;14(8):e0218298. doi: 10.1371/journal.pone.0218298 (PMC6711513; doi:10.1371/journal.pone.0218298)
Supplement: S1 Table — (DOCX) [file pone.0218298.s006.docx]

**S1 Table. Comparison of mRNA levels in the intervertebral disc (IVD) between passive smoking and non-smoking rats**

| Group | Gene | 🡹🡻^a^ | *p*-value | | Comparison^b^ | TaqMan^c^ |
| --- | --- | --- | --- | --- | --- | --- |
|  |  |  | N4:S4 | N8:S8 |  |  |
| Apoptosis  (Death receptor pathway) | *Fasl* | 🡹 |  |  |  | Rn00563754_m1 |
|  | *Fas* | 🡹 |  |  |  | Rn00685720_m1 |
|  | *Flip* | 🡻 |  |  |  | Rn00589205_m1 |
| Apoptosis  (Mitochondria pathway) | *TP53* | 🡹 |  |  |  | Rn00755717_m1 |
|  | *Bax* | 🡹 |  |  |  | Rn02532082_g1 |
|  | *Noxa* | 🡹 |  |  |  | Rn01494552_m1 |
|  | *Puma* | 🡹 | 0.251 | 0.175 | N>S | Rn00597992_m1 |
|  | *JNK1* | 🡹 |  |  |  | Rn01453358_m1 |
|  | *JNK2* | 🡹 |  |  |  | Rn00569058_m1 |
|  | *Bcl-2* | 🡻 | 0.676 | 0.175 | N<S | Rn99999125_m1 |
|  | *Dusp1* | 🡻 | 0.347 | 0.175 | N<S | Rn00678341_g1 |
|  | *Hsp 70* | 🡻 |  |  |  | Rn00583013_s1 |
|  | *14-3-3* | 🡻 |  |  |  | Rn00695953_m1 |
| Extracellular matrix | *Acan* |  |  |  |  | Rn00573424_m1 |
|  | *Col2a1* |  |  |  |  | Rn00563954_m1 |
| Extracellular matrix‐degradation | *Mmp13* |  | 0.748 | 0.109 | N<S | Rn01448195_m1 |
|  | *Mmp3* |  |  |  |  | Rn00591740_m1 |
|  | *Adamts4* |  | 0.045 | 0.078 | N>S | Rn02103282_s1 |
| Apoptosis  (Others) | *Vdr* | 🡹 |  |  |  | Rn00566976_m1 |
|  | *Runx2* | 🡻 | 0.423 | 0.150 | N<S | Rn01512298_m1 |
|  | *Hif-1α* | 🡹🡻 | 0.092 | 0.337 | N>S | Rn00577560_m1 |
|  | *Xiap* | 🡻 |  |  |  | Rn0147299_m1 |

The mRNA levels of the apoptosis-related genes type II collagen, aggrecan and degrading enzymes were measured. Expression of genes involved in the transcriptional regulation of apoptosis-related genes, such as *Vdr, Runx2* and *Hif-1α,* was also analyzed. Comparisons between groups were made using the Mann-Whitney U-test and those with a *p*-value <0.2 are shown here. RNA was extracted from the nucleus pulposus (NP) and annulus fibrosus (AF) without the cartilage end-plate (CEP), and subjected to expression analysis as described in S2 Figure. Physiological apoptosis was noted, but no passive cigarette smoking-induced changes in apoptosis were evident (S3 and S4 Figs). However, changes in expression were noted in some apoptosis-related genes, although these changes were not significant. These molecules are involved in both the promotion and inhibition of apoptosis, which may have been the reason for the absence of changes in apoptosis.

^a^Upward and downward arrows represent known up-regulation and down-regulation in apoptosis, respectively.

^b^N<S, increased expression in the passive smoking group; N>S, decreased expression in the passive smoking group. N4, non-smoking control for 4 weeks; S4, passive smoking for 4 weeks; N8, non-smoking control for 8 weeks; S8, passive smoking for 8 weeks.

^c^TaqMan Gene Expression Assay ID (Applied Biosystems, Foster, USA) used for quantitative PCR.
