# Supplementary material for: Detection of apoptosis and matrical degeneration within the intervertebral discs of rats due to passive cigarette smoking
Source: PLoS One. 2019 Aug 27;14(8):e0218298. doi: 10.1371/journal.pone.0218298 (PMC6711513; doi:10.1371/journal.pone.0218298)
Supplement: Attachment — Submitted filename: Response to Reviewers.docx [file pone.0218298.s007.docx]

July 26, 2019

Manuscript ID PONE-D-19-14928
Title: **Detection of apoptosis and matrical degeneration within the intervertebral discs of rats due to passive cigarette smoking.**

Author: Masahiro Nakahashi et al.

Dear Dr. Joerg Heber, Editor-in-Chief, PLOS ONE

:

We appreciate the time and efforts by the editor and referees in reviewing this manuscript. We also thank the reviewers for valuable comments and suggestion. We have addressed all issues indicated in the review report, and believed that the revised version can meet the journal publication requirements. Please find our point-by-point responses to the editor’s and reviewer’s comments and the revised manuscript highlighting the revisions made.

In accordance with the editor’s comment, we attached our revised Competing Interests Statement here:

This work was funded in part by Ono Pharmaceutical Co., Ltd., Stryker Japan K. K. and Nakashima Medical Co., Ltd. received by YT. There are no patents, products in development or marketed products associated with this research to declare. This does not alter our adherence to PLOS ONE policies on sharing data and materials.

Sincerely yours,

Mariko Esumi

Department of Biomedical Sciences

Nihon University School of Medicine

Ohyaguchikami-cho, Itabashi-ku

Tokyo 173-8610, Japan

Phone: +81-3-3972-8111 Ext. 2241

Fax: +81-3-3972-8199

E-mail: esumi.mariko@nihon-u.ac.jp

**Point-by-point responses to editor’s and reviewer’s comments:**

To Editor

**Comment 1:**

*Please ensure that your manuscript meets PLOS ONE's style requirements, including those for file naming.*

**Response:**

We have reviewed the PLOS ONE style templates and corrected our manuscript.

**Comment 2:**

*We note that you received funding from a commercial source: "Ono Pharmaceutical Co., Ltd" and "Nakashima Medical Co., Ltd"*

*Please provide an amended Competing Interests Statement that explicitly states this commercial funder, along with any other relevant declarations relating to employment, consultancy, patents, products in development, marketed products, etc.*

*Within this Competing Interests Statement, please confirm that this does not alter your adherence to all PLOS ONE policies on sharing data and materials by including the following statement: "This does not alter our adherence to PLOS ONE policies on sharing data and materials.”*

**Response:**

We have corrected the Competing Interests Statement as follows:

This work was funded in part by Ono Pharmaceutical Co., Ltd., Stryker Japan K. K. and Nakashima Medical Co., Ltd. received by YT. There are no patents, products in development or marketed products associated with this research to declare. This does not alter our adherence to PLOS ONE policies on sharing data and materials.

*Please include your amended Competing Interests Statement within your cover letter. We will change the online submission form on your behalf.*

**Response:**

We have included our amended COI statement within our cover letter.

**Comment 3:**

*Please note that all PLOS journals ask authors to adhere to our policies for sharing of data and materials: https://journals.plos.org/plosone/s/data-availability. According to PLOS ONE’s Data Availability policy, we require that the minimal dataset underlying results reported in the submission must be made immediately and freely available at the time of publication. As such, please remove any instances of 'unpublished data' or 'data not shown' in your manuscript and replace these with either the relevant data (in the form of additional figures, tables or descriptive text, as appropriate), a citation to where the data can be found, or remove altogether any statements supported by data not presented in the manuscript.*

**Response:**

In accordance with the editor’s comment, we have revised the **Introduction on page 3, line 67** by adding a **reference 27 (**written in Japanese) **on page 19, lines 477-480**. We have also revised **Fig 2B** by adding a graph of AF; accordingly, we have revised the **Result on page 8, line 192-194** and **Fig 2B legend on page 8, lines 213 and 214.**

**Comment 4:**

*To comply with PLOS ONE submissions requirements, in your Methods section, please provide additional information on the animal research and ensure you have included details on the frequency of welfare monitoring and any efforts to alleviate suffering.*

**Response:**

In accordance with the editor’s comment, we have revised the **Materials and Methods on page 4, lines 84-88, and lines 93-94.**

To Reviewer #1

**Comment 1:**

*After euthanasia, the spine samples were harvested and then fixed in formalin or frozen at -80°C. The authors should consider doing the perfusion (first with saline to remove blood cells and followed by formalin to preserve the antigen). After perfusion, the samples would not contain the blood cells, which may affect the immunohistochemical staining.*

**Response:**

In order to obtain the intact RNA and exactly analyze the mRNA expression, we did not perform the perfusion. Instead, we performed cardiac puncture for blood removal, and quickly excised the entire spine. IVD is　avascular tissue by nature, and there was no problem about the immunohistochemical staining in our experiment.

*By the way, what are the samples frozen at -80°C use for? Are these samples for mRNA measurement? The authors should clarify.*

**Response:**

Yes, the samples frozen at -80°C were used for mRNA and protein analysis. In accordance with the reviewer’s comment, we have revised the **Materials and Methods on page 4, lines 96 and 97.**

**Comment 2:**

*The authors found that type II collagen and aggrecan were decreased in the NP and CEP, while the synthesis of these two proteins were not affected by the smoking, as reflected by the mRNA repression measurement. How did the authors measure the mRNA? The authors should state the specific methods in the Methods section.*

**Response:**

In accordance with the reviewer’s comment, we have added the section ‘Quantification of mRNA’ in the **Materials and Methods on page 4-5, lines 103-110.** We have also added a **reference 30 on page 19, lines 490-494.**

**Comment 3:**

*The evidence about the passive cigarette smoking-induced apoptosis in the CEP was not strong. The authors should add some other experiments, such as TUNEL staining. Also, the authors found that no significant changes in apoptosis-related gene expression were observed in the NP or AF in response to passive cigarette smoking. What about the protein expression? they should consider measuring the related protein expression via immunohistochemical staining.*

**Response:**

We agree with the reviewer that other apoptosis assessments are necessary to confirm the apoptosis of the CEP. Actually, we tried to assess the apoptosis of the CEP by cleaved caspase-3 staining and TUNEL method. Unfortunately, the two methods did not work in our system, even in the positive control specimen, the thymus of rats treated with dexamethasone. It seems to be due to the tissue preparation such as fixation in our experiment. Therefore, we have not done the immunohistochemistry of other apoptosis-related protein. Only the ssDNA staining worked in the positive control tissue and was used here.

Thus, considering the limitation, we dampened the statement and discussed on the apoptosis of CEP, followed by several supportive data of other groups.

**Comment 4:**

*In the conclusion, the authors stated passive cigarette smoking-induced stress stimuli first affect the CEP through blood flow. However, there was no results reflecting the blood flow. How did they prove that the blood flow was affected?*

**Response:**

As the reviewer pointed out, we have no evidence for the reduction of blood flow in our experiment. We have revised the Abstract on page 2, line 36: from ‘concluded’ to ‘hypothesized’.

However, there have been several reports on blood flow reduction in the IVD induced by cigarette smoke and nicotine. We have revised the **Discussion on page 13, lines 331-334 and 337** by citing these reports (**references 38** and 18)**.** We have also added a **reference 38 on page 21, lines 524-527.**
